# Temporary epicardial left ventricular and biventricular pacing improves cardiac output after cardiopulmonary bypass

**DOI:** 10.1186/1749-8090-7-113

**Published:** 2012-10-22

**Authors:** Jose B García-Bengochea, Angel L Fernández, Daniel Sánchez Calvelo, Julian Alvarez Escudero, Francisco Gude, José R González Juanatey

**Affiliations:** 1 Cardiac Surgical Division. Department of Surgery, Hospital Clínico Universitario, 15706, Santiago de Compostela, Spain; 2 Department of Anesthesiology, Santiago de Compostela, Spain; 3 Epidemiological Clinic, Santiago de Compostela, Spain; 4 Department of Cardiology, Hospital Clínico Universitario, 15706, Santiago de Compostela, Spain

**Keywords:** Postoperative cardiac pacing, Left ventricular, Biventricular, Cardiac output optimization, Atrial fibrillation

## Abstract

**Background:**

To evaluate, with different pacing modes, acute changes in left ventricular systolic function, obtained by continuous cardiac output thermodilution in various subsets of patients undergoing cardiopulmonary bypass surgery. Increments of mean arterial pressure and cardiac output were considered the end point.

**Methods:**

Fifty cases electively submitted to cardiac surgery were analyzed. Isolated valve surgery 62%, coronary revascularization 30% and 8% mixed disease. Left ventricular ejection fraction was preserved in 50%,36% had moderate depression,(EF 36%-50%) whereas 14% had severe depression (EF < 35%). Left bundle branch block occurred in 18%. Preoperatively 84% were in sinus rhythm and 16% in atrial fibrillation. The different subgroups were analyzed for comparisons. Right atrial-right ventricular and right atrial-left ventricular pacing were employed in sinus rhytm. Biventricular pacing was also used in atrial fibrillation.

**Results:**

Right atrium-right ventricular pacing, decreased significantly mean arterial pressure and cardiac output (2.3%) in the overall population and in the subgroups studied. Right atrium-left ventricle, increased mean arterial pressure and cardiac output in 79% of patients and yielded cardiac output increments of 7.5% (0.40 l/m) in the low ejection fraction subgroup and 7.3% (0.43 l/m) in the left bundle branch block subset. In atrial fibrillation patients, left ventricular and biventricular pacing produced a significant increase in cardiac output 8.5% (0.39 l/min) and 11.6% (0.53 l/min) respectively. The dP/dt max increased significantly with both modes (p = 0.021,p = 0.028).

**Conclusion:**

Right atrial-right ventricular pacing generated adverse hemodynamic effects. Right atrium-left ventricular pacing produced significant CO improvement particularly in cases with depressed ventricular function and left bundle branch block. The greatest increments were observed with left ventricular or biventricular pacing in atrial fibrillation with depressed ejection fraction.

## Background

The benefits of cardiac re-synchronization therapy (CRT) in chronic heart failure with severe left ventricular (LV) dysfunction due to ventricular dyssynchrony have been established by several randomized controlled trials [1-3], demonstrating that the use of left ventricular pacing (LVP) or biventricular (BiVP)) pacing improves cardiac function by generating a more efficient ventricular contraction. Besides, deleterious effects of chronic right ventricular (RV) apical pacing on LV performance have been reported [4].

Cardiac function is often diminished after cardiopulmonary bypass (CPB). The acute ventricular asynchrony and systolic dysfunction generated during this period, far exceed those seen in the setting of chronic heart failure (CHF) treated by CRT. Delayed recovery of myocardial performance following restoration of myocardial flow, referred as “stunning”, is a well documented phenomenon, associated to CPB reperfusion [5].

The adverse movement of the interventricular septum toward the right ventricle, during systolic contraction it is another common finding after CPB [6]. The above mentioned alterations, and a depressed preoperative ventricular function, are often responsible of hemodynamic (HD) instability, which difficult weaning of CPB, requiring inotropic and vasoactive support, electrical stimulation and even mechanical assistance.

Placement of RV temporary epicardial electrodes, is a routine procedure during cardiac surgery to treat bradicardia with low cardiac output (CO) or atrio-ventricular block. This pacing site, usually generates an undesirable cardiac effect, due to the PSM created by the initial RV activation [7]. Cannesson et al. [8] have shown, that acute RA- RVP after CPB, in the absence of right bundle branch block (RBBB), worsens CO.

Nelson et al. [9], have demonstrated that LVP or BiVP can acutely improve systolic function in cases with intraventricular conduction delay, without increasing myocardial oxygen extraction (MV02). This new therapeutic pathway has been investigated by Bakhtiary and co-.authors [10],finding that BiVP was associated with improved LV contractility without rising MV02 compared with atrial pacing This fact may allow to diminish inotropic administration protecting additionally myocardial metabolism. Therefore optimization of temporary stimulation (TS) after CPB,particularly in cases with depressed LV function it must be considered a coadyuvant method for the improvement of CO, which at certain perioperative stages may be crucial.

At present, and despite recent clarifying investigations concerning pacing configurations, stimulation site, perioperative phase and simultaneous adjustments of atrioventricular (AVD) and interventricular delays (VVD) [11-14], there is still absence of clear recomendations to optimized TS. This fact, is mainly due to the above mentioned variables employed in heterogeneous populations and in a changeable and complex scenario.

Our initial experience [15] using biventricular (BiV) dual cathodal stimulation which allows VVD adjustments, promoted the present prospective controlled clinical trial. The aim of this study was to assess the potential hemodynamic benefits of TS, employing routine pacing configurations and sites during the immediate postoperative phase in a wide sample of patients. Improvement of CO, an important short-term parameter, was considered the end point.

## Methods

### Patient population

A total of 50 patients electively submitted to CPB surgery between March 2008 and May 2010 were included in the present study. Institutional ethical committee approval was obtained. All patients gave fully written informed consent. Exclusion criteria were the presence of previous pacemaker, severe tricuspid incompetence in order to maintain the accuracy of thermodilution derived CO measurements and intracardiac shunt. The development of cardiac arrhythmias in the early post-operative period, requiring antiarrhythmic drugs or postoperative low cardiac output needing mechanical support were also considered an exclusion criteria

Mean age was 67.14 ± 11.18 years (range 39–82). Preoperative diagnosis included, isolated valve disease in 31 cases (62%), isolated coronary artery disease in 15 cases (30%) and mixed disease (8%) requiring valve surgery with coronary artery bypass grafting (CABG) (Table 1).

**Table 1:** Demographic, preoperative and intraoperative characteristics of the population included in the study

**Number**	**(n) 50**
Male/female	38/12
Age (years)	67.14 ± 11.18
LVEF (%)	
≥50%	50
35-49%	36
<35%	14
Isolated valve disease (%)	62% (31)
Isolated coronary artery disease (%)	30% [15]
Mixed valve and coronary disease (%)	85 [4]
Sinus rhythm (%)	84
Atrial fibrillation (%)	16
LBBB (%)	18
RBBB (%)	4
Median sternotomy approach	100%
Aortic cross clamp time (min)	73 ± 29.5
Cardiopulmonary bypass time (min)	91 ± 21.5
Warm intermittent cardiopegia	100%

LVEF: Left ventricular ejection fraction. LBBB: Left bundle branch block. RBBB: Right bundle branch block.

Data are expressed as mean ± SD.

Left bundle branch block (LBBB) was present in 9 patients (18%) and RBBB in 2 (4%). Left ventricular ejection fraction (LVEF) derived from transthoracic Doppler echocardiography using the Simpson method was preserved (LVEF ≥ 50%) in 25 cases (50%); moderate depression (LVEF 3%-49%) in 18 cases (36%), and severe dysfunction (LVEF < 35%) in 7 cases (14%). Sinus rhythm (SR) was present in 84% of cases and 16% were in atrial fibrillation (AF) with depressed LVEF.

Intraoperative patient monitoring included invasive blood pressure and continuous ECG registry. Following general anesthesia and orotracheal intubation, a heparin coated Swan-Ganz catheter (Opti-Q,Abbott Critical Care System, Abbott Laboratories, IL,USA) was placed for hemodynamic management and determinations: pulmonary artery pressures and systemic vascular resistance. The catheter was connected to a monitor (Q2 CCO/SvO2 computer, Abbot Lab, IL,USA) for continuous monitoring of CO and mixed venous oxygen saturation (SvO2). The use of validated automated CO method allows determinations of rapid change in CO, useful for modifying pacing parameters: atrio-ventricular delay (AVD), interventricular delay (VVD) and pacing configurations. Surgery was performed through a median sternotomy and under standard CPB. After aortic cross clamping, cold hyperkalemic antegrade blood cardioplegia was administered for cardiac arrest and repeated every 20 min. Controlled reperfusion was achieved using a single dose of warm cardioplegia. After completion of the surgical procedure, weaning of CPB and removal of atrial canulae, epicardial unipolar pacing wires (Streamline 6500; Medtronic, Inc, Minneapolis, Minn. USA) were placed at the right atrial appendage (RA), diaphragmatic surface of the RV (DSRV) and posterobasal LV wall, then passed percutaneously and sutured to the skin at the lower end of the sternotomy incision.

In all cases, basal hemodynamic parameters: CO, systolic, diastolic and mean arterial pressure (MAP), pulmonary artery pressures, SvO2 and a 12 lead surface electrocardiogram were registered, 1–2 h after admission to the intensive care unit and once the patient had reached hemodynamic stability. Patients were sedated and mechanical ventilation parameters were held constant during the study. Norepinephrine was administered if required in order to reach MAP of at least 60 mmHg. No other inotropic agents were administered during the procedure. Blood pressures were determined as the mean of the values obtained at the end of three consecutive respiratory cycles. During the study, volume administration or intravenous drug infusions were held constant, whitout additional medications. Cases with a post-CPB heart rate > 120 beats/min were excluded. Stimulation was performed at a rate 10% superior to the basal rate during 6 min, using the different configurations and parameters. Then discontinued for 10 min after each period. External temporary dual-chamber generators (model 5345, Medtronic Inc, Minneapolis, Min, USA and ERA 300 Biotronik, SE&Co, Berlin,Germany) were employed.

In AF cases, for achievement of dual cathodal BiVP with VVD adjustments, an implantable pulse generator, Biotronik Logos D, was used as an external pacemaker. The atrial port of the generator was usually connected to the LV lead and the ventricular port to the RV electrode, using the generator as the positive pole to close the circuit. In patients in SR two configurations were used: a) RA-LV bipolar anocathodal mode (cathode in LV). b) RA-RV bipolar anocathodal mode (cathode in RV)The AVD was modified within a range of 80 to 200 ms, according to the patients heart rate, MAP and CO monitoring. In AF patients, three different configurations were used :a) LV bipolar anocathodal (cathode in LV, anode in RV). b) RV bipolar anocathodal (cathode in RV, anode in LV).c) BiVP dual cathodal (cathodes in LV and RV) with adjustable VVD. With QRS <120 mm the delays ranged from +20-40 ms. With a wide QRS > 120 mm, the delays ranged from 0–80 ms.At least 3 settings were used in each study. The highest CO obtained was considered the optimal.

Echocardiographic assessment was only performed in AF cases. Mitral regurgitation jets were imaged using colour Doppler echocardiography in parasternal long-axis and four chamber view. The maximal regurgitant jets were determined during intrinsic rhythm and with the various pacing configurations in order to calculate the dP/dt max as a contractility index. Left ventricular dyssynchrony was assessed using tissue Doppler imaging by transthoracic or transesophageal echo-doppler ultrasound.

Analysis of the whole population was obtained. Several sub-groups were established for comparisons according to: LVEF, type of BBB, basal rhythm and type of surgery. Subjects served as their own control.

Statistical analysis was performed with the Wilcoxon rank sum test for paired data, using SPSS software (SPSS, Inc., Illinois, USA). Due to pairewise comparisons, Bonferroni correction was performed. A p-value of less than 0.05 was considered statistically significant. Continuous data were expressed as mean ± standard deviation.

## Results

The analysis of the overall population comparing RA-LVP and RA-RVP modes with basal values showed a significant increase in CO and MAP in favour of RA-LVP, close to 80% of patients, whereas RA-RV, significantly decreased both parameters (Table 2).

**Table 2:** Analysis of the hemodynamic parameters of the overall population

**Hemodynamic parameters**	**Basal**	**Stimulation mode**	
		**RA-LVP**	**RA-RVP**
HR (bpm)	72,80 ± 8,60	83,70 ± 4,22**	83,74 ± 4,74**
SAP (mmHg)	107,80 ± 14,75	111,24 ± 18,88**	98,76 ± 15,08**
DAP (mmHg)	52,64 ± 7,59	55,50 ± 8,32**	49,96 ± 8,10**
MAP (mmHg)	71,01 ± 6,2	74,4 ± 6,8**	66,4 ± 6,1**
CO (L/min)	5,30 ± 1,54	5,61 ± 1,72*	5,08 ± 1,60**
Sv02 (%)	69,96 ± 7,10	69,80 ± 8,01	68,28 ± 7,81

RA-LVP: Right atrium-left ventricle. RA-RVP: Right atrium-right ventricle. HR (bpm): Heart rate beats per minute. SAP (mmHg): Systolic arterial pressure. DAP (mmHg): Diastolic arterial pressure. MAP (mmHg): Mean arterial pressure. CO (L/min): Cardiac output. SvO2 (%): Mixed venous oxygen saturation. *p < 0.05;** p < 0.01.

Data are expressed as mean ± SD.

In the subgroup with preserved LVEF, RA-LVP pacing produced a significant increase of CO, although not of MAP. With RA-RVP, a significant decrease of CO and MAP was noticed (table 3). In the low EF sub-group, the RA-LVP, increased significantly CO and MAP with increments of 7.5% (0.40 l/m) and 7.7% respectively. (Table 3).

**Table 3:** Analysis in patients with preserved left ventricular ejection fraction and depressed left ventricular ejection fraction, including moderate and severe dysfunction

**Hemodynamic parameters**	**Basal**	**Preserved left ventricular function (n = 25)**	
		**RA-LVP**	**RA-RVP**
HR (bpm)	72,80 ± 8,28	83,88 ± 4,55**	84,04 ± 4,62**
SAP (mmHg)	111,28 ± 16,29	109,80 ± 21,46*	100,32 ± 16,08*
DAP (mmHg)	57,76 ± 7,76	56,16 ± 8,69*	50,96 ± 9,12*
MAP (mmHg)	75,5 ± 14,2	74,1 ± 21,1	67,4 ± 17,6*
CO (L/min)	5,30 ± 1,37	5,52 ± 1,65*	4,93 ± 1,36*
Sv02 (%)	69,56 ± 7,62	69,28 ± 8,46	67,96 ± 8,72
		Depressed left ventricular function (n = 25)	
HR (bpm)	72,80 ± 8,28	83,60 ± 4,55**	83,44 ± 4,38**
SAP (mmHg)	104,32 ± 12,31	112,68 ± 14,48*	97,20 ± 14,17*
DAP (mmHg)	50,52 ± 6,93	54,84 ± 8,09*	48,96 ± 6,98*
MAP (mmHg)	68,4 ± 3,7	74,1 ± 5,9**	65,7 ± 4,9**
CO (L/min)	5,30 ± 1,72	5,70 ± 1,81*	5,11 ± 1,84**
Sv02 (%)	70,36 ± 8,28	70,32 ± 7,67	68,60 ± 6,95

RA-LVP: Right atrium-left ventricle. RA-RVP: Right atrium-right ventricle. HR (bpm): Heart rate beats per minute. SAP (mmHg): Systolic arterial pressure. DAP (mmHg): Diastolic arterial pressure. MAP (mmHg): Mean arterial pressure. CO (L/min): Cardiac output. SvO2 (%): Mixed venous oxygen saturation. *p < 0.05;** p < 0.01.

Data are expressed as mean ± SD.

The LBBB subgroup, showed that RA-LVP increased significantly CO and MAP: 7.3% (0.43 l/min) and 8.9% respectively. In contrast, RA-RVP decreased significantly both (Table 4). Also in the subgroup without LBBB, RA-LVP increased both CO and MAP (Table 4).

**Table 4:** Analysis in the group of patients with and without left bundle branch block

**Hemodynamic parameters**	**Basal**	**With LBBB (n = 9)**	
		**RA-LVP**	**RA-RVP**
HR (bpm)	74,33 ± 7,48	84,22 ± 3,23**	84,22 ± 3,23**
SAP (mmHg)	99,11 ± 7,38	109,22 ± 15,25*	89,11 ± 12,10**
DAP (mmHg)	51,56 ± 7,38	55,44 ± 9,04*	47,33 ± 5,02**
MAP (mmHg)	67,3 ± 5,2	73,3 ± 6,1*	61,3 ± 4,5*
CO (L/min)	5,56 ± 1,81	5,97 ± 2,09**	5, 23 ± 1,90*
Sv02 (%)	68,22 ± 7,22	69,00 ± 7,54	67,00 ± 5,98
		Without LBBB (n = 41)	
HR (bpm)	72,46 ± 8,87	83,63 ± 4,44**	83,63 ± 4,72**
SAP (mmHg)	109,71 ± 15,88	111,68 ± 18,90*	100,08 ± 14,96**
DAP (mmHg)	52,88 ± 7,70	55,51 ± 8,27*	50,54 ± 8,57**
MAP (mmHg)	71,8 ± 4,3	74,2 ± 4,8	67,0 ± 5**
CO (L/min)	5,25 ± 1,49	5,54 ± 1,67*	4,97 ± 1,55*
Sv02 (%)	70,34 ± 7,11	65,95 ± 8,19	68,56 ± 8,19

RA-LVP: Right atrium-left ventricle. RA-RVP: Right atrium-right ventricle. HR (bpm): Heart rate beats per minute. SAP (mmHg): Systolic arterial pressure. DAP (mmHg): Diastolic arterial pressure. MAP (mmHg): Mean arterial pressure. CO (L/min): Cardiac output. SvO2 (%): Mixed venous oxygen saturation. LBBB: Left bundle branch block. *p < 0.05;** p < 0.01.

Data are expressed as mean ± SD.

In the valve surgery sub-group, RA-LVP, improved significantly CO (5.14 ± 1.47 l/m vs 5.44.1.71 ± l/m; p < 0.05) and MAP (72.6 ± 4.1 mmHg vs 75.7 ± 5 mmHg; p < 0.0.01) with regard to basal values. With RA-RVP, CO significantly decreased (5.14 ± 1.47 l/m vs 4.831,43 ± l/m; p < 0.05) and also MAP (72.6 ± 4.1 mmHg vs 67.8 ± 6.1 mmHg; p < 0.001).

In the CABG sub-group, only CO increased significantly with RA-LVP (5.66 ± 1.68 l/m vs 5.99 ± 1.76 l/m, p < 0.05), whereas RA-RVP significantly decreased CO (5.66 ± 1.68 l/m vs 5.47 ± 1.97 l/m; p < 0.05) and MAP (69.6 ± 3.8 mmHg vs 64.3 ± 4.5 mmHg, p < 0.001) relative to basal values. The combined CABG and valve surgery sub-group, showed an increase of MAP (66.6 ± 5.2 mmHg vs 71.4 ± 4.4 mmHg) and CO (5.20 ± 1.74 l/m vs 5.47 ± 1.83 l/m) with RA-LVP and a decline of CO (5.20 ± 1.74 l/m vs 4.77 ± 1.4 l/m) with RA-RVP, without statistical significance (4 cases only).

In the SR group with RA-LVP, CO significantly increased (5.5%) and also MAP (Table 5).

**Table 5:** Hemodynamic parameters in the group of patients with sinus rhythm (n = 42)

**Hemodynamic parameters**	**Basal**	**Stimulation mode**	
		**RA-LVP**	**RA-RVP**
HR (bpm)	73,74 ± 8,66	83,93 ± 4,51**	83,98 ± 4,75**
SAP (mmHg)	106,81 ± 14,38	108,67 ± 15,33*	97,38 ± 14,03**
DAP (mmHg)	52,36 ± 7,92	54,14 ± 7,51*	50,00 ± 8,32**
MAP (mmHg)	70,4 ± 3,1	72,1 ± 3,4**	65,8 ± 4,3**
CO (L/min)	5,44 ± 1,57	5,74 ± 1,77*	5,16 ± 1,65*
Sv02 (%)	69,74 ± 6,62	69,17 ± 7,11	67,83 ± 7,22

RA-LVP: Right atrium-left ventricle. RA-RVP: Right atrium-right ventricle. HR (bpm): Heart rate beats per minute. SAP (mmHg): Systolic arterial pressure. DAP (mmHg): Diastolic arterial pressure. MAP (mmHg): Mean arterial pressure. CO (L/min): Cardiac output. SvO2 (%): Mixed venous oxygen saturation. *p < 0.05;** p < 0.01.

Data are expressed as mean ± SD.

In the AF group, LVP and BiVP, yielded a significant increase of CO:8.5% (0.39 l/min) and 11.6% (0.53 l/min) respectively, without significant differences between them (Table 6).

**Table 6:** Hemodynamic parameters in the group of patients with atrial fibrillation (n = 8)

**Hemodynamic parameters**	**Basal**	**LVP**	**RVP**	**BiVP**
HR (bpm)	67,88 ± 6,77	82,75 ± 2,12**	82,50 ± 4,38**	82,64 ± 2,16**
SAP (mmHg)	113,00 ± 16,36	124,75 ± 26,90*	106,00 ± 19,21**	126 ± 24,89
DAP (mmHg)	54,13 ± 5,74	62,63 ± 9,18*	49,75 ± 7,38**	60,14 ±8,16
MAP (mmHg)	72,6 ± 3,5	83,1 ± 4,2*	68,4 ± 4*	85,30 ± 11,31*
CO (L/min)	4,57 ± 1,19	4,96 ± 1,30*	4,30 ± 1,18*	5,10 ± 1,78*
Sv02 (%)	67,88 ± 6,77	73,13 ± 11,75*	70,63 ± 10,09	74,0 ± 14,68*

Stimulation mode.

BiV: Biventricular dual cathodic. RA-LVP: Right atrium-left ventricle. RA-RVP: Right atrium-right ventricle. HR (bpm): Heart rate beats per minute. SAP (mmHg): Systolic arterial pressure. DAP (mmHg): Diastolic arterial pressure. MAP (mmHg): Mean arterial pressure. CO (L/min): Cardiac output. SvO2 (%): Mixed venous oxygen saturation. *p < 0.05;** p < 0.01.

Data are expressed as mean ± SD.

No significant chnages in Sv02, were found in the overall population nor in any of the subgroups, except in the AF group, which increased significantly with LVP and BiVP (Table 6). No significant differences in pulmonary artery pressures were observed.

**Figure 1:**
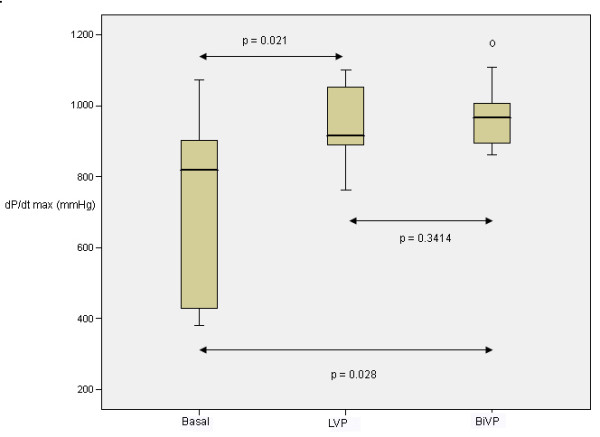
Boxplot representation of dP/dt max (mmHg/s) values in atrial fibrillation patients using left ventricular pacing (LVP) and biventricular pacing (BiVP) related to basal values.

Echocardiographic evaluation of this group, showed lateral wall septal dyssynchrony (SS-PPS delay >60 ms) in all cases. LVP and BIVP produced symmetrical contraction of the LV mid-septal and lateral wall with both pacing modes. The .dP/dt max,(Figure 1) increased significantly with both LVP and BiVP, without significant differences between them The highest increments of dP/dt max where obtained in cases with the lowest basal values.

## Discussion

Temporary stimulation on the RVDS with epicardial electrodes after CBP is a common practice in cardiac surgery. Different studies, investigating the sites of pacing to optimize CO including: RVP at, apical, diaphragmatic surface, paraseptal and outflow tract; LVP: posterobasal, mid free-wall, apical and paraseptal, have generated controversial results [16-21].

Vaughan et al. [22] performed an extreme search on the subject. They concluded that only 9 of the 13 publications, resulted in significant increases of cardiac index,up to 22% with BiVP or AR-LVP. Tanaka et al. and others [18,22,23] observed greatest benefits in patients with low EF and wide QRS complex. Whereas others, report no significant hemodynamic improvement with these pacing modes or even no adverse effects with RA-RVP in populations with normal ventricular function [19-21].

The strategy for perioperative optimization, by means of LVP or BiVP using a LV basal cathodal electrode and adequate adjustments of AVD and VVD if feasible, is gaining progressive acceptance, to improve CO perioperatively, particularly in cases with depressed EF and LBBB [11,13,18,23]

In our study, in an heterogeneous population, RA-LVP,LVP or BiVP effectiveness, was validated by MAP and CO monitoring, proving to be beneficial in almost 80% of the whole population, with significant improvements of MAP and CO in the low EF and LBBB subgroups, being highest in the AF group with depressed EF. The increments obtained may appear rather modest but simultaneously avoid the adverse effects of RVP. Similar positive results with RA-LVP have been obtained by Flynn et al. [16].

In the average population, RA-RVP decreases CO and MAP and this effects also occurs in the different subgroups studied. Similar adverse observations have been reported in cases undergoing CABG surgery [8,18]. Besides, it has been noticed that with low EF, systolic dyssynchrony with RA-RVP is significantly higher compared with RA-LVP or BiVP [13],except in cases with RBBB [18]. From our study, no conclusions can be reached as there were only two cases with such alteration.

Our investigation, shows a 20% of non responders. They were tested only by RA-RVP or RA-LVP modes with AVD adjustments. The most delayed site on the inferolateral LV wall was not established by echocardiography and BiVP was not applied in the SR group, lacking a possible optimization of synchronicity with VVD.

In the setting of postoperative CPB, there is very limited experience reported with AF, generally due to the established exclusion criteria. In an acute HD study in CHF, including AF cases, Blanc et al. [24] observed a significant increase of systolic blood pressure with both LVP and BiVP.

Mixed venous oxygen saturation, only showed favourable significant differences, in the group of AF with EF < 35%, using LVP or BiVP. Eberhardt el al [17] in CABG patients, did not found differences in SvO2 among the various pacing modalities. Our results seem to indicate that SvO2, may not be a suitable parameter to validate the effectiveness of the different postoperative TS modes, when ventricular function is preserved.

Due to the nonexistence of an external triple chamber pacemaker, we employed only in AF cases, a biventricular dual cathodal pacing system [15], which implies two independent activated circuits, with a cathode in each ventricle, allowing VVD adjustments. To our knowledge the present study, using dual cathodal BiVP is the only clinical experience reported, in cases with AF, during the postoperative CPB period.

Few reports provide a precise description of the BiVP configuration used, accomplished either with the split bipole or the dual cathodal split system, but always with the drawback of not been able to adjust the VVD, to maximize the optimization benefit [11,12]. Fernandez et al. (30) questioned the assessment of the potential hemodynamic benefits of TS, based on the fact, that different authors have used distinct terminology for the pacing configurations of left anocathodal system, versus biventricular left cathodal split bipole.

Our study,in AF cases with EF < 35%, both LVP and BiVP significantly increased MAP and CO, accompanied by significant improvements of dP/dt max, without statistical differences between them. Flynn et al. [16] in a subset of five cases of AF submitted to CABG, placing an active lead on the LV posterobasal area, did not observed significant changes in MAP.

A recent study [12] under acute RV and LV failure conditions, has demonstrated using BiVP, that the dP/dt max of the failing ventricle, is maximized when interventricular contraction is close to synchronous. During acute ventricular failure, BiVP parameters like LVP site and the correct VVD, can recruit the unstressed ventricle to support function of the failing one by “interventricular assist”.

Right acute ventricular failure after CPB is an important hemodynamic complication difficult to treat effectively. BiVP with VVD adjustments, could be very helpful in that setting stressing the relevance of this pacing mode.

Wang et al. [11] in a substudy of the BiPACS trial (mean LVEF < 35%), have reported an increase of 14% in CO after AVD optimization compared with the worst value and 7% mean increase from an AVD of 120 ms. The optimum VVD differed from the nominal value, in 5% CO improvement. Overall, optimized BiVP resulted in a CO increase of 10% versus SR. Schmidt and co-workers [19],pointed out the limitation of not using AVD and VVD optimization with BiVP, after not obtaining any HD improvement in CBPG cases.

These results, further stress the relevance of optimizing AVD and VVD in the perioperative CPB setting, particularly, in cases with preexisting LV dysfunction, at high risk of developing acute low-output state. Nevertheless, the mechanisms by which pacing optimization improves hemodynamics in this setting, are still not fully defined [14] and require further dedicated studies.

Future investigations using TS to improve cardiac function, will be more feasible using external triple chamber generators with adjustable VVD and should contribute to establish pacing optimization as a routine step of perioperative protocols.

## Conclusion

The present study revealed that for the overall population and the different subgroups analyzed, RA-RVP at the diaphragmatic site induces a decrease of MAP and CO.

Overall, 79% of the heterogeneous population analyzed, improved CO and MAP using RA-LVP, BiVP or LVP. In the preserved LVEF subgroup, there was an increase in CO of 4.15%. The benefits increased in the LBBB subgroup reaching 6.6%. With depressed LVEF there was a further increase up to 7.5%. The highest increase, 11.6% was obtained with BiVP in AF cases with depressed LVEF.

Non responders to RA-LVP, should be tested by BiVP with VVD adjustments and with the accompaniment of individualized echocardiographic assessment.

### Limitations of the study

Mechanisms underlying the absence of positive response to RA-LVP in 20% of the sample remain unknown. Individual ecochardiographic assessment in SR cases was not performed and the most delayed activated site on the LV wall not determined. In addition, in these subjects BiVP was not applied.

## Competing interests

The authors declare that they have no competing interests.

## Authors’ contribution

JBGB, ALF, and DSC have made substantial contributions to conception and design or acquisition of data, or analysis and interpretation of data.

JAE, FG and JRGJ have been involved in drafting the manuscript and revising it critically.

All authors have given final approval of the version to the be published.
